# Biochemical Characterization of a First Fungal Esterase from *Rhizomucor miehei* Showing High Efficiency of Ester Synthesis

**DOI:** 10.1371/journal.pone.0077856

**Published:** 2013-10-30

**Authors:** Yu Liu, Haibo Xu, Qiaojuan Yan, Shaoqing Yang, Xiaojie Duan, Zhengqiang Jiang

**Affiliations:** 1 Bioresource Utilization Laboratory, College of Engineering, China Agricultural University, Beijing, China; 2 Department of Biotechnology, College of Food Science and Nutritional Engineering, China Agricultural University, Beijing, China; Montana State University, United States of America

## Abstract

**Background:**

Esterases with excellent merits suitable for commercial use in ester production field are still insufficient. The aim of this research is to advance our understanding by seeking for more unusual esterases and revealing their characterizations for ester synthesis.

**Methodology/Principal Findings:**

A novel esterase-encoding gene from *Rhizomucor miehei* (*RmEstA*) was cloned and expressed in *Escherichia coli*. Sequence analysis revealed a 975-bp ORF encoding a 324-amino-acid polypeptide belonging to the hormone-sensitive lipase (HSL) family IV and showing highest similarity (44%) to the *Paenibacillus mucilaginosus* esterase/lipase. Recombinant RmEstA was purified to homogeneity: it was 34 kDa by SDS-PAGE and showed optimal pH and temperature of 6.5 and 45°C, respectively. The enzyme was stable to 50°C, under a broad pH range (5.0–10.6). RmEstA exhibited broad substrate specificity toward *p-*nitrophenol esters and short-acyl-chain triglycerols, with highest activities (1,480 U mg^−1^ and 228 U mg^−1^) for *p*-nitrophenyl hexanoate and tributyrin, respectively. RmEstA efficiently synthesized butyl butyrate (92% conversion yield) when immobilized on AOT-based organogel.

**Conclusion:**

RmEstA has great potential for industrial applications. RmEstA is the first reported esterase from *Rhizomucor miehei*.

## Introduction

Esterase (carboxylesterase, EC 3.1.1.1) and lipase (EC 3.1.1.3) are usually known as lipolytic enzymes catalyzing the cleavage and formation of ester bonds. Esterases mainly catalyze the hydrolysis of ester bonds of short-chain triglycerides or esters (<10 carbon atoms), whereas lipases favor the hydrolysis of water-insoluble triglycerides bearing long-chain fatty acids (>10 carbon atoms) [1]. Under certain conditions (anhydrous organic solvent systems), both types of enzymes can catalyze the reverse reaction, esterification, as well as transesterification and enantioselective hydrolysis reactions [2]. The most significant difference between esterases and lipases is in their ability to act on surface display: lipases exhibit interfacial activation whereas esterases do not [3].

Due to their unique properties, such as a broad range of non-natural substrate specificity, high stability in organic solvents and high enantioselectivity (for some of them), esterases are useful for a variety of industrial applications: as additives in fat and oil processing, in laundry detergents, for the synthesis of fine chemicals and pharmaceuticals, in paper making and in the manufacture of cosmetics, among others [2]–[4]. In the food industry in particular, they exhibit numerous potential applications in flavor-ester producing processes, since flavorings represent over a quarter of the world market for food additives, consumers have been shown to prefer foodstuff that can be labeled as “natural”, and the enzyme-based biochemically produced flavor esters excel, with better odors and flavors than their chemical counterparts [5]. To date, various flavor esters have been synthesized by esterification reaction using microbial lipolytic enzymes [5], [6]. However, esterases seem to be less popular than lipases, mainly due to lack of availability despite their commercial value. Accordingly, identification, isolation and characterization of novel esterases with unique properties are of great value for application in the industry.

Esterases are widely distributed in various mammalian tissues, plants and microorganisms [7]. Microbial esterases have gained considerable attention from the industry, because they are more stable and much easier to produce on a large scale. So far, a number of microbial esterases have been identified and characterized from various microbes, including *Bacillus subtilis* [8], *B. licheniformis* [9], *Rhodococcus* sp. LKE-028 [10] and *Pleurotus sapidus* [11]. Many bacterial esterase genes have been cloned and expressed, such as from *Pseudoalteromonas* sp. 643A [4], *Oenococcus oeni* [12], *P. arctica* [13], *Thermoanaerobacter tengcongensis* [14], *Pseudomonas putida* [15] and *Geobacillus thermodenitrificans* T2 [16]. Several esterase genes have also been isolated and cloned from metagenomic libraries such as fermented compost [17], soil [18], and intertidal flat sediment [19]. To date, few investigations have been reported on fungal esterases than on those obtained from bacterial or mammalian sources [20]–[22].

*Rhizomucor miehei* is a thermophilic zygomycete that secretes multiple hydrolytic enzymes, such as lipase [2], milk clotting protease [23] and β-glucanase [24]. However, no esterases have been characterized from this species. In this paper, we describe the first molecular cloning and expression of a novel esterase gene from *R. miehei* CAU432 and characterize the recombinant enzyme. In addition, the potential for application of this esterase to flavor-ester synthesis was investigated.

## Materials and Methods

### Materials

PrimeSTAR HS DNA polymerase and restriction endonucleases were purchased from TaKaRa (Tokyo, Japan). T4 DNA ligase was obtained from New England Biolabs (Ipswich, MA, USA). Linalyl acetate, linalool, *p*-nitrophenol (*p*NP), *p*-nitrophenyl acetate (*p*NPA), *p*-nitrophenyl butyrate (*p*NPB), *p*-nitrophenyl caprylate (*p*NPC), *p*-nitrophenyl decanoate (*p*NPD), *p*-nitrophenyl laurate (*p*NPL), *p*-nitrophenyl myristate (*p*NPM), *p*-nitrophenyl palmitate (*p*NPP), 4-methylumbelliferyl butyrate and Fast Red TR Salt were purchased from Sigma Chemical Co. (St. Louis, MO, USA). *p*-Nitrophenyl hexanoate (*p*NPH) was obtained from HEOWNS Company (Tianjin, China). Triacetin, tributyrin, tricaproin, tricaprylin and tricaprin were obtained from TCI Co. (Tokyo, Japan). All other chemicals were of analytical grade unless otherwise stated.

### Strains and Plasmids

The *Rhizomucor miehei* strain CAU432 used in this study has been deposited in the China General Microbiological Culture Collection Center (CGMCC, accession No. 4967). *E. coli* strain DH5α was used to propagate plasmids and strain BL21 (DE3) was used as the host for expression of the esterase gene. pET-30a(+) was obtained from Novagen (Madison, WI, USA). pMD18-T was purchased from TaKaRa.

### Cloning of the Full-length Esterase cDNA and Sequence Analysis

DNA manipulations were performed as described by Sambrook and Russell [25]. Genomic DNA was isolated from *R. miehei* CAU432 mycelia using the CTAB (Hexadecyltrimethy Ammonium Bromide) method. For mycelium collection, *R. miehei* CAU432 was cultivated at 50°C for 2 days in medium containing (g L**^−1^**): oat flour 10, tryptone 10, yeast extract 10, MgSO_4_·7H_2_O 0.3, FeSO_4_ 0.3 and CaCl_2_ 0.3, then the fungal mycelia were collected by centrifugation (5,000×*g*, 10 min) and washed twice with sterilized water at 4°C. Total RNA was isolated using the Trizol Kit (Invitrogen, Carlsbad, CA, USA), and mRNAs were purified using the Oligotex mRNA Midi Kit (Qiagen, Dusseldorf, Germany).

Genomic DNA of *R. miehei* CAU432 was used as the template for subsequent polymerase chain reaction (PCR) amplification. To clone the esterase gene, degenerate primers EstDF and EstDR (Table 1) were designed based on the conserved blocks of amino acid residues (LAVAGDSAG and DVLRDEGE) from other known esterases using the CODEHOP algorithm [26]. A putative homologous consensus region of the esterase gene was amplified using the degenerate primers and analyzed by sequencing the PCR products. PCR conditions were as follows: a hot start at 94°C for 5 min followed by 10 cycles of 94°C for 30 s, 60°C for 30 s and 72°C for 1 min, with 0.5°C decrease in annealing temperature per cycle, then 20 cycles of 94°C for 30 s, 55°C for 30 s and 72°C for 1 min. The PCR product was purified, ligated to pMD18-T vector, transformed into *E. coli* DH5α competent cells and sequenced.

**Table 1:** Primers used in this study.

Primers	Primer sequence (5′→3′)
EstDF^a^	CCGTCGCCGGCgaywsngcngg
EstDR^a^	CTCGCCCTCGTCTcknarnacrtc
Est5′GSP	ATCCGATTCAGCAGTGAATAAAAGA
Est5′NGSP	AGCAGTGAATAAAAGAGCTGG
Est3′GSP	GGAGCTACATTGTCTGCTGCAGTA
Est3′NGSP	TACATTGTCTGCTGCAGTATCC
EstDNAF	ATGACTGTCGGAAACCCACCAA
EstDNAR	TTATGCATTATACTTTGCATAAATGTCACG
RmEstAF^b^	GGGTTTCATATGACTGTCGGAAACCCACCAA
RmEstAR^b^	ATTCCGCTCGAGTGCATTATACTTTGCATAAATGTCACG

a Y = C/T, W = A/T, S = C/G, N = A/T/C/G, K = G/T, R = A/G.

b Restriction enzyme sites incorporated into primers are underlined.

The full-length cDNA sequence of the esterase was obtained by 5′ and 3′ rapid amplification of cDNA ends (RACE) using a BD SMART™ RACE cDNA Amplification Kit (Clontech, Palo Alto, CA, USA). The PCR conditions for RACE were as follows: a hot start at 94°C for 1 min, followed by 30 cycles of 30 s at 94°C, 30 s at 68°C and 1 min at 72°C, and finally 10 min at 72°C. PCR was performed with the following primer pairs (Table 1): Est5′GSP and Universal Primer A Mix for the first PCR, followed by a nested PCR with the primers Est5′NGSP and Nested Universal Primer A for 5′ RACE. Similarly, 3′ RACE was performed with Est3′GSP and Universal Primer A Mix, followed by nested PCR with Est3′NGSP and Nested Universal Primer A. The obtained PCR product was purified, cloned and sequenced. The 5′ and 3′ flanking sequences obtained by 5′ and 3′ RACE were assembled with that of the consensus region to form the full-length cDNA sequence containing the open reading frame (ORF) of the esterase gene. To amplify this region from the genomic DNA of *R. miehei* CAU432, the same PCR conditions were used with the specific primers EstDNAF and EstDNAR (Table 1). The purified PCR product was ligated with pMD18-T vector and transformed into *E. coli* DH5α for sequencing.

Nucleotide and deduced amino-acid sequences were analyzed using DNAMAN software. BLAST analysis was performed at the NCBI server (http://blast.ncbi.nlm.nih.gov/Blast.cgi). Amino-acid sequences were aligned using the ClustalW program (ftp://ftp-igbmc.u-strasbg.fr/pub/ClustalW/). Signal peptide was analyzed by SignalP 4.0 server (http://www.cbs.dtu.dk/services/SignalP/). Analysis of conserved domain and signature sequences was carried out using ScanProsite (http://prosite.expasy.org/scanprosite/). *N*-Glycosylation sites were predicted using NetNGlyc1.0 (http://www.cbs.dtu.dk/services/NetNGlyc/).

### Expression of the Esterase Gene in *E. coli*

The ORF encoding the esterase (designated RmEstA) was amplified by PCR from the cDNA of *R. miehei* CAU432 with primers RmEstAF and RmEstAR (Table 1). *Nde*I and *Xho*I sites (underlined) were added to the forward and reverse primers, respectively, and the expressed protein carried a C-terminal His tag encoded by the vector. PCR conditions were as follows: a hot start at 94°C for 5 min, 30 cycles of 94°C for 30 s, 55°C for 30 s and 72°C for 1 min, followed by one cycle of 72°C for 10 min. The purified PCR product was digested by *Nde*I and *Xho*I, subcloned into the similarly digested pET-30a(+) vector, and transformed into *E. coli* BL21 competent cells for protein expression. A single colony of *E. coli* BL21 harboring RmEstA in pET-30a(+) was inoculated into LB medium containing kanamycin (50 µg mL^−1^) and incubated on a rotary shaker (200 rpm, 37°C) until the optical density at 600 nm (OD_600_) reached about 0.6–0.8. Isopropyl β-D-thiogalactopyranoside (IPTG) was added to a final concentration of 1 mM, and the culture was then grown at 30°C for 12 h in a rotary shaker at 200 rpm.

### Purification of the Recombinant Esterase (RmEstA)

The cells (from 1 L of culture) were harvested by centrifugation and homogenized in 10 mL buffer A (50 mM pH 8.0 Tris-HCl buffer containing 500 mM NaCl and 30 mM imidazole) and disrupted by ultrasonication. Cell debris was removed by centrifugation at 8,000×*g* for 20 min at 4°C. The supernatant was loaded onto a Ni-IDA column (1 cm×5 cm) pre-equilibrated with buffer A. After the column had been washed with 15 column volumes (CV) of buffer A followed by 5 CV of buffer B (50 mM Tris-HCl pH 8.0 containing 500 mM NaCl and 50 mM imidazole), the bound proteins were eluted with buffer C (50 mM Tris-HCl pH 8.0, 500 mM NaCl, 200 mM imidazole). The elution rates were all adjusted to 1.0 mL min^−1^. The protein fractions with esterase activity were pooled and concentrated, and the buffer was exchanged (50 mM phosphate buffer pH 6.5) using a 10-kDa MW cut-off ultrafiltration membrane.

### Enzyme Assay and Protein Determination

Esterase activity was determined spectrophotometrically using *p*NPH as the substrate according to the method of Sumby et al. [12] with minor modifications: 50 µL of suitably diluted enzyme was prepared in 400 µL of 50 mM phosphate buffer (pH 6.5) and after preheating for 2 min, 50 µL of 20 mM *p*NPH substrate (in pure isopropanol) was added. The mixture was incubated at 45°C for 10 min, and then 500 µL of 300 mM sodium phosphate buffer (pH 7.0) containing 5% (w/v) SDS was added. The liberated *p*NP was quantified by measuring the absorbance at 410 nm. One unit of enzyme activity was defined as the amount of enzyme required to liberate 1 µmol *p*NP per minute under the above conditions. The assay of interfacial activation was performed according to the methods of Martinelle et al. [27].

Protein concentration was measured by the method of Lowry et al. [28] using bovine serum albumin as the standard. Specific activity was expressed as units per milligram protein.

### SDS-PAGE and Zymogram Analysis

SDS-PAGE was performed according to the method of Laemmli [29] using 4.5% stacking gel and 12.5% separating gel. The protein bands were stained with Coomassie brilliant blue R-250. A low molecular weight calibration kit (GE Healthcare) containing phosphorylase b (97.0 kDa), bovine serum albumin (66.0 kDa), ovalbumin (45.0 kDa), carbonic anhydrase (30.0 kDa), trypsin inhibitor (20.1 kDa) and α-lactalbumin (14.4 kDa) was used. Esterase activity staining was performed as described by Karpushova et al. [30].

### Effect of pH and Temperature on the Activity and Stability of the Purified Esterase

The effect of pH on enzyme activity was determined by measuring esterase activity in different buffers (50 mM) from pH 2.5 to 10.6 using the standard enzyme assay. The following buffers were used: glycine-HCl (pH 2.5–3.5), citrate buffer (pH 3.0–6.0), 2-(N-morpholino)ethanesulfonic acid (MES) buffer (pH 5.5–6.5), phosphate buffer (pH 6.0–8.0), Tris-HCl buffer (pH 7.5–9.0) and glycine-NaOH (pH 8.6–10.6). To determine its pH stability, the purified enzyme was incubated in the listed buffers at 45°C for 30 min, and then residual activity was measured at 45°C in 50 mM sodium phosphate buffer (pH 6.5).

The optimal temperature for the purified esterase’s activity was examined by measuring the enzyme activity at temperatures ranging from 30°C to 80°C in 50 mM phosphate buffer (pH 6.5). Thermostability of the esterase was investigated by incubating the enzyme (in 50 mM pH 6.5 phosphate buffer) at various temperatures (30–80°C) for 30 min. Then the residual activities of the samples withdrawn at different time intervals were measured at 45°C in 50 mM phosphate buffer (pH 6.5).

The effect of metal ions on the enzyme stability was evaluated by incubating the enzyme (40 µg mL^−1^) in 50 mM phosphate buffer (pH 6.5) at 30°C for 1 h in the presence of 10 mM various metal ions. Then the residual activity was measured.

### Substrate Specificity and Kinetic Parameters

Substrate specificity of the purified esterase toward *p*NP esters was determined using *p*NPA, *p*NPB, *p*NPH, *p*NPC, *p*NPD, *p*NPL, *p*NPM and *p*NPP as the substrates. The reaction mixture was the same as described in the enzyme assay, except that it contained 0.1% (v/v) Triton X-100, 0.1% (w/v) arabic gum and different substrates. After incubating at 45°C for 10 min, the reaction was stopped by adding 0.5 mL chilled stop reagent (300 mM sodium phosphate buffer pH 7.0 containing 5% SDS), the mixture was centrifuged at 12,000×*g* for 3 min, and the absorbance of the supernatant was immediately measured at 410 nm [12]. Substrate specificity for synthetic triacylglycerols (triacetin, tributyrin, tricaproin, tricaprylin and tricaprin) and for olive oil was analyzed titrimetrically at 45°C for 10 min using 10 mM NaOH for titration according to the method of Eggert et al. [8]. The substrates at 10 mM (8.72 g L^−1^ for olive oil) were emulsified in 20 mL reaction buffer containing 2.5 mM phosphate buffer (pH 6.5) and 1% arabic gum. The reaction was initiated with the addition of 10 µL purified enzyme, and NaOH consumption was recorded. One unit of enzyme activity was defined as the amount of enzyme releasing 1.0 µmol fatty acid per minute.

The kinetic parameters of the purified esterase toward *p*NPA, *p*NPB, *p*NPC, *p*NPD, *p*NPH and *p*NPL were determined by measuring the enzyme activities with different substrate concentrations in 50 mM sodium phosphate buffer (pH 6.5) at 45°C for 5 min. The constant kinetic parameters of *K*_m_ and *V*_max_ were calculated using “GraFit” software.

### Synthesis of Butyl Butyrate using Immobilized RmEstA

The purified RmEstA was immobilized on sodium bis(2-ethylhexyl) sulfosuccinate (AOT)-based organogels as described by Dandavate and Madamwar [31]. The immobilized RmEstA was used to synthesize butyl butyrate. The synthetic reaction was performed in 10 mL of mixture containing butyric acid, 1-butanol and immobilized esterase at 45°C for 7 days in an orbital shaker with rotation speed of 150 rpm. A 50-µL aliquot of the reaction mixture was withdrawn every 24 h and immediately analyzed by gas chromatography. Gas chromatography analysis was performed using an Agilent GC model 7890A, equipped with a Varian CP-Dex CB capillary column (25 m×0.25 mm×0.25 µm) and a flame ionization detector (FID) according to the method of De Barros et al. [32]. In all cases, hydrogen was used as carrier gas. The injector and detector temperature were set at 200°C and 250°C, respectively. The oven temperature was raised to 150°C at a rate of 15°C per minute after it was held at 50°C for 4 min.

To determine the effect of substrate concentration and ratio on esterification efficiency, the concentration of one substrate was held constant while that of the other was varied. In the first set of reactions, butyric acid concentration was held constant at 100 mM and that of 1-butanol was varied from 50 to 200 mM. In the second set of reactions, butyric acid concentration was varied from 50 to 200 mM while that of 1-butanol was held constant at the optimal concentration obtained in the first experiment.

## Results

### Cloning and Sequence Analysis of the Esterase Gene from *R. miehei*

**Figure 1:**
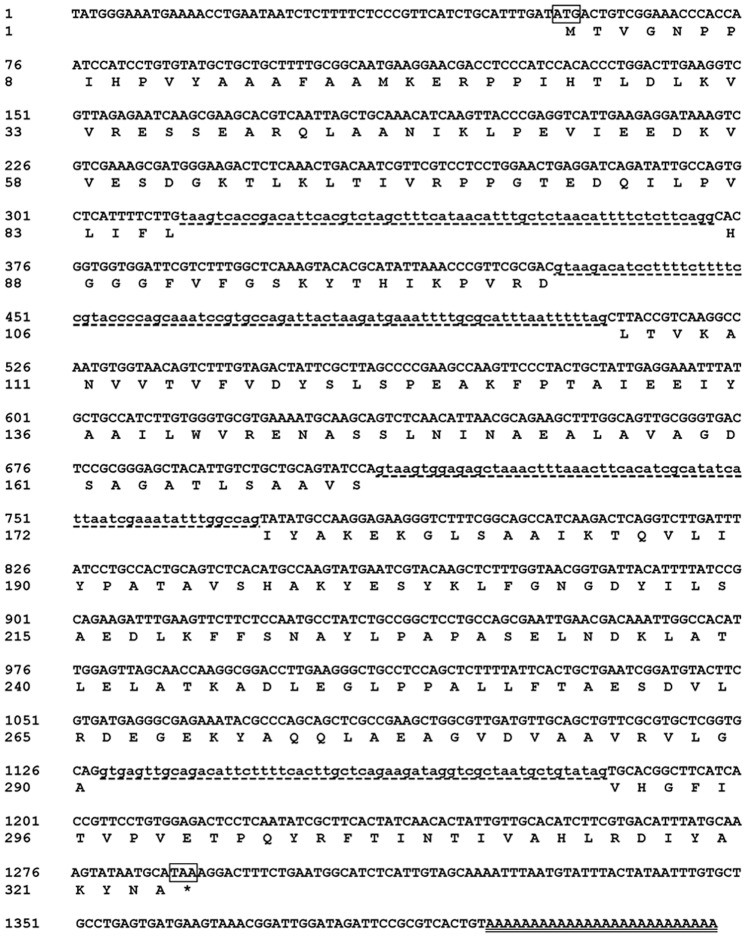
Nucleotide and deduced amino acid sequences of the cDNA, flanking regions and genomic DNA of RmEstA. Conceptual translation of the ORF to the 324 amino acids is shown in one-letter code below the respective codon. The translation initiation codon ATG and termination codon TAA are boxed. An asterisk indicates the stop codon. Four intron sequences are shown in lowercase letters with dotted underlining. The poly(A+) tail is double-underlined. The nucleotide sequence reported here was submitted to GeneBank and given the accession number KC310704.

Based on the conserved amino-acid sequences of known esterase genes, a 407-bp fragment from *R. miehei* was amplified with degenerate primers (Table 1). The fragment shared highest nucleotide sequence identity (50%) with the esterase gene from *Herbaspirillum* sp. CF444 (EJL92844). Thus, this partial sequence was used to design gene-specific primers for 5′ and 3′ RACE, to obtain the sequence of the full-length gene. The 5′ and 3′ RACE yielded 861-bp and 646-bp DNA fragments, respectively. The two flanking regions were then assembled with the core fragment to generate a 1,162-bp cDNA sequence containing a putative full-length ORF (*RmEstA*) of 975 bp. Comparison with a 1,235-bp genomic sequence indicated the presence of four introns of 60, 81, 62 and 57 bp in the coding region. The nucleotide and deduced amino-acid sequences of the full-length cDNA and flanking regions of the *RmEstA* gene are shown in Figure 1. The deduced protein consisted of 324 amino-acid residues with a predicted molecular mass of 34,989 Da and a theoretical p*I* of 5.23. The N-terminal region contained no predicted signal peptide, and the protein sequence did not contain any potential *N*-glycosylation sites. The sequence of *RmEstA* has been submitted to GenBank with accession number of KC310704.

**Figure 2:**
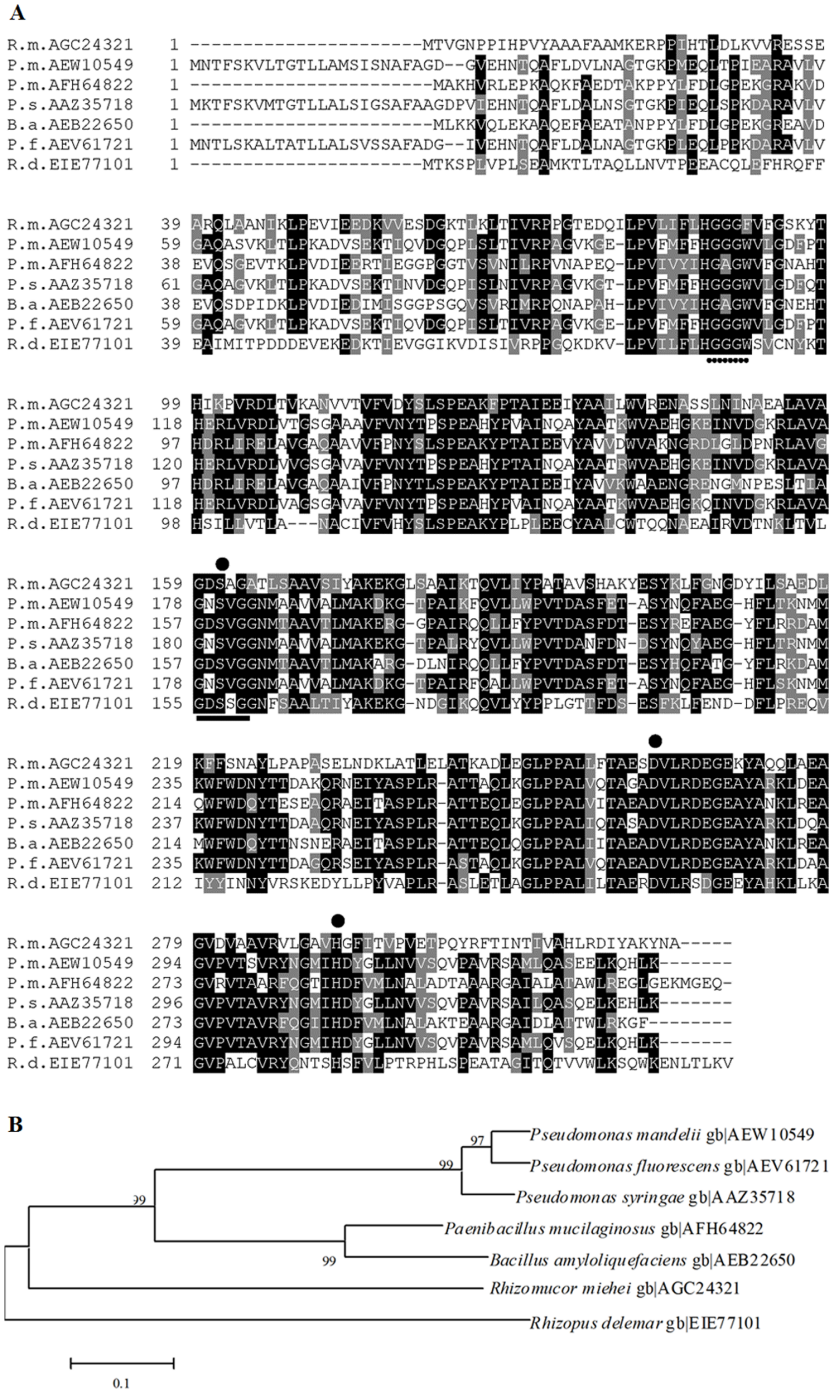
Multiple alignment (A) and phylogenetic dendrogram (B) of the representative esterases. (A) Multiple alignment of esterase amino acid sequences. Numbers on the left are the residue number of the first amino acid in each line. Abbreviations and accession numbers for the esterases are as follows: *Rhizomucor miehei* (R.m. AGC24321), *Pseudomonas mandelii* (P.m. AEW10549), *Paenibacillus mucilaginosus* (P.m. AFH64822), *P. syringae* (P.s. AAZ35718), *Bacillus amyloliquefaciens* (B.a. AEB22650), *P. fluorescens* (P.f. AEV61721) and *Rhizopus delemar* (R.d. EIE77101). Identical residues are shaded in black, and conserved residues are shaded in gray. The conserved catalytic motif is underlined. The conserved HSL family motif is shown with dotted underlining. The putative catalytic nucleophile and acid/base are identified by a filled circle. (B) Phylogenetic dendrogram based on full-length amino-acid sequences of esterases by neighbor-joining algorithm, showing the position of RmEstA from *R. miehei* CAU432 relative to other esterases. The dendrogram is shown with the microbial sources and GenBank accession numbers of the esterases. Bootstrap values are expressed as percentages of 1,000 replications. Bar = 0.1 sequence divergence.

According to the sequence similarity results from the BLAST search, the amino-acid sequence similarities of RmEstA to esterases/lipases from the bacteria *Pseudomonas mandelii* (AEW10549), *Paenibacillus mucilaginosus* (AFH64822), *P. syringae* (AAZ35718), *Bacillus amyloliquefaciens* (AEB22650) and *P. fluorescens* (AEV61721) were 44%, 43%, 42%, 42% and 41%, respectively; RmEstA shared only 43% identity with a putative esterase (EIE77101) from the fungus *Rhizopus delemar* (Figure 2A). A phylogenetic tree comprising all of these members was constructed (Figure 2B). Results showed that RmEstA is much more closely related to the fungal esterase (*R. delemar* gb|EIE77101) than to those from bacteria, thus indicating that it is a novel fungal esterase. BLAST homology search of the sequence of RmEstA identified it as belonging to the α/β hydrolase superfamily. RmEstA contained the esterase-conserved catalytic nucleophile S161 in the consensus pentapeptide GDSAG. The other two amino acids of the enzyme’s catalytic triad were predicted as D262 and H292. A conserved HGGG motif was found upstream of the active-site conserved motif from amino acid 87 to 90, which indicated that RmEstA belongs to the hormone-sensitive lipase (HSL) family (family IV) [18], [33].

Nucleotide sequencing of *RmEstA* revealed a 975-bp ORF encoding a protein of 324 amino acids. The sequence of RmEstA contained the typical conserved esterase/lipase motif, GXSXG (Figure 1). Multiple amino-acid-sequence alignment indicated that the present esterase sequence shares relatively low identity (less than 44%) with those of other reported bacterial esterases, and 43% identity with only one putative fungal esterase from *Rhizopus delemar* (EIE77101), indicating that RmEstA should be considered a novel member of the esterases.

### Expression and Purification of the Recombinant Esterase (RmEstA)

**Figure 3:**
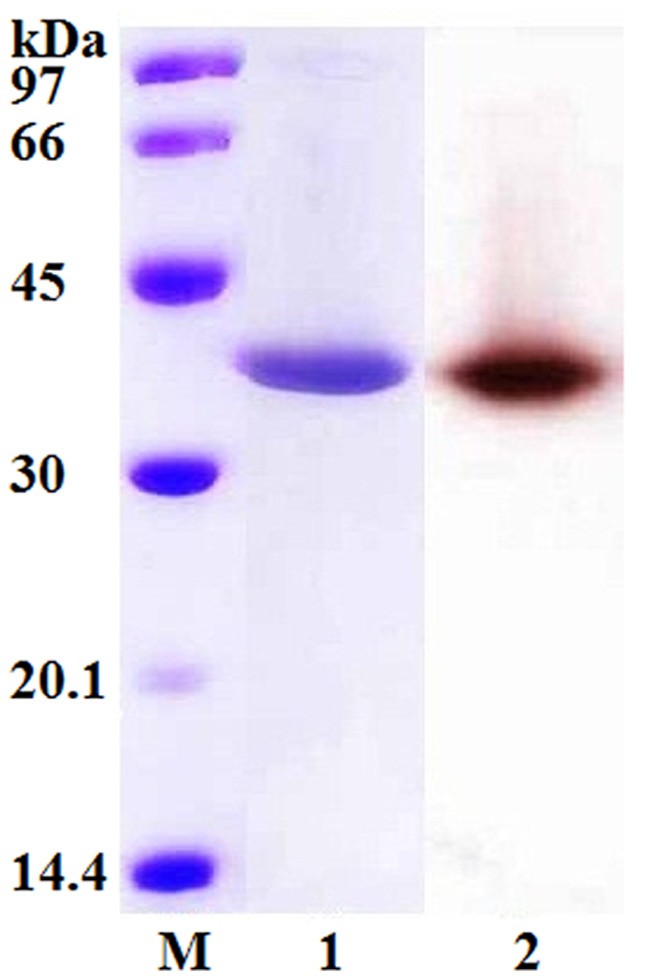
SDS-PAGE and zymogram analysis of the purified RmEstA from *R. miehei.* Lane M, low-molecular-weight protein standards; lane 1, purified esterase (RmEstA); lane 2, zymogram analysis of esterase on SDS-PAGE indicated by α-naphthyl acetate.

*RmEstA* was expressed in *E. coli* BL21 as a soluble intracellular enzyme. RmEstA was purified to homogeneity by Ni-IDA chromatography with 12-fold purification and an overall yield of 75%. The specific activity of RmEstA was increased to 1,480 U mg^−1^ (data not shown). The purified enzyme migrated on an SDS-polyacrylamide gel as a single homogeneous band of 34 kDa, matching the molecular mass (34,989 Da) calculated from the deduced amino-acid sequence (Figure 3).

### Effect of pH and Temperature on the Activity and Stability of RmEstA

**Figure 4:**
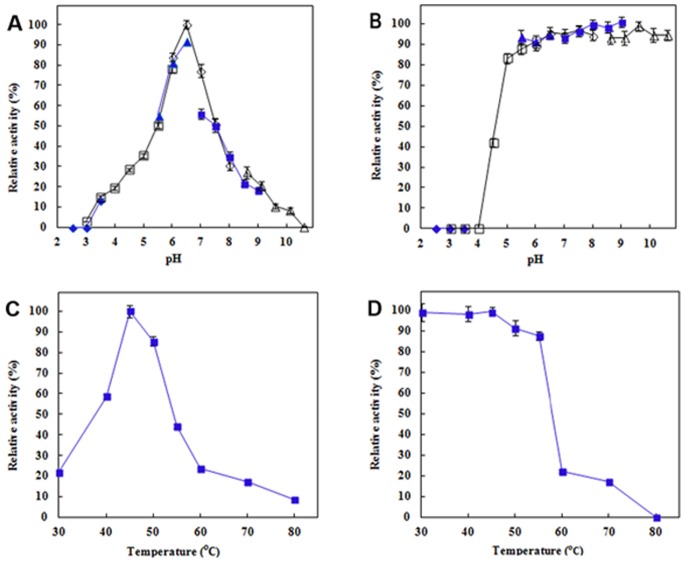
Optimal pH (A), pH stability (B), optimal temperature (C) and thermostability (D) of RmEstA from *R. miehei*. Buffers used in (A) and (B) were (♦) Glycine-HCl (pH 2.5–3.5), (□) sodium citrate (pH 3.0–6.0), (▴) MES buffer (pH 5.5–6.5), (◊) sodium phosphate buffer (pH 6.0–8.0), (▪) Tris-HCl buffer (pH 7.5–9.0) and (△) Glycine-NaOH buffer (pH 8.6–10.6).

RmEstA exhibited optimal activity at pH 6.5 in 50 mM phosphate buffer (Figure 4A). The enzyme was fairly stable over a broad range of pHs (5.0–10.6), retaining more than 90% of its activity (Figure 4B). The optimal temperature for RmEstA activity was 45°C (Figure 4C), and it was stable at up to 50°C, with more than 90% of its activity remaining (Figure 4D). The thermal denaturing half life of the enzyme at 40, 55 and 60°C was 219, 156 and 15 min, respectively (data not shown).

The influence of metal ions on enzyme stability is presented in Table 2. The esterase’s activity was strongly inhibited by Cu^2+^, Mg^2+^, Fe^2+^, Ag^+^, Hg^2+^, but only moderately inhibited by Sn^2+^. In contrast, the enzyme was markedly activated by Zn^2+^, Mn^2+^, Co^2+^ and Ba^2+^, and moderately activated by Na^+^ and Cr^2+^. No significant effect of Ca^2+^and Ni^2+^ was observed on enzyme activity.

**Table 2:** Effect of metal ions on RmEstA activity.

Metal ions	Specific activity (U mg^−1^)	Residual activity (%)^a^
Control	1480	100
Cu^2+^	610±21	41±1.4
Ca^2+^	1480±52	100±3.5
Ni^2+^	1550±62	104±4.2
Zn^2+^	2000±25	135±1.7
Mg^2+^	890±21	60±1.4
Mn^2+^	1830±56	124±3.8
Co^2+^	3340±92	225±6.2
Na^+^	1640±36	111±2.4
Ba^2+^	2210±62	149±4.2
Hg^2+^	87±2.9	5.9±0.2
Cr^2+^	1760±56	119±3.8
Fe^2+^	540±19	36±1.3
Ag^2+^	280±5.9	1.9±0.4
Sn^2+^	1260±34	85±2.3

a Esterase was incubated at 30°C for 1 h in 50 mM phosphate buffer (pH 6.5) in the presence of 10 mM metal ions. The residual activity was then measured by standard enzyme assay. Values represent the means of three replicates ± standard error.

### Substrate Specificity and Kinetic Parameters of RmEstA

Specific activities of RmEstA on different substrates are shown in Table 3. The enzyme hydrolyzed *p*NP derivatives with acyl-chain lengths ranging from C_2_ to C_16_; the highest hydrolysis rate was observed with *p*NPH (1,480 U mg^−1^), followed by *p*NPB (1250 U mg^−1^), *p*NPC (850 U mg^−1^) and *p*NPD (380 U mg^−1^). RmEstA showed relatively low activity on various emulsified synthetic triglycerides, displaying its highest activity of 228 U mg^−1^ with tributyrin as the substrate. When the carbon chain length of the substrate increased to above 4 or decreased, the specific activity declined dramatically (Table 3). RmEstA displayed no interfacial activation using *p*NPB as no sharp increase of enzyme activity was observed when the concentration of substrate increased from 0 to 4 mM (form soluble to insoluble) (data not shown). The Michaelis-Menten constant (*K*_m_) for *p*NPA (C_2_), *p*NPB (C_4_), *p*NPH (C_6_), *p*NPC (C_8_), *p*NPD (C_10_) and *p*NPL (C_12_) was determined to be 1.0, 0.17, 0.12, 0.82, 0.28 and 0.3 mM, respectively (Table 4).

**Table 3:** Substrate specificity of RmEstA from *R. miehei*.

Substrate	Chain length	Specific activity (U mg^−1^)	Relative activity (%)
*p*NP esters^a^			
*p*NPA	C_2_	370±5.2	24
*p*NPB	C_4_	1250±24	84
*p*NPH	C_6_	1480±36	100
*p*NPC	C_8_	850±11	57
*p*NPD	C_10_	380±6.9	25
*p*NPL	C_12_	150±4.2	10
*p*NPM	C_14_	20±0.8	1.5
*p*NPP	C_16_	6±0.2	0.4
Triglycerides^b^			
Triacetin	C_2_	76±2.5	26
Tributyrin	C_4_	228±6.1	100
Tricaproin	C_6_	79±1.9	35
Tricaprylin	C_8_	69±1.2	30
Tricaprin	C_10_	53±0.8	23
Olive oil		15±0.4	6

a Assays with *p*NP esters were performed at 45°C in 50 mM phosphate buffer (pH 6.5) containing 0.1% (v/v) Triton X-100 and 0.1% (w/v) arabic gum. Values are shown relative to the highest observed activity, which was arbitrarily designated 100%.

b Activities with olive oil and synthetic triglycerides as substrates were measured in 2.5 mM phosphate buffer (pH 6.5) containing 0.1% (v/v) substrate and 0.1% (w/v) arabic gum.

**Table 4:** Kinetic parameters of RmEstA^a^.

Substrate	*V*_max_(*µ*mol/min mg)	*K*_m_ (mM)	*k*_cat_ (s^−1^)	*k*_cat_/*K*_m_(mM^−1^ s^−1^)
*p*NPA	643	1	0.37	0.37
*p*NPB	2018.4	0.17	1.17	6.9
*p*NPH	1852.6	0.12	1.08	8.97
*p*NPC	721	0.82	0.42	0.51
*p*NPD	62.3	0.28	0.04	0.13
*p*NPL	33	0.3	0.019	0.06

a The kinetic parameters of the purified esterase were determined by measuring the enzyme activities with different substrate concentrations in 50 mM sodium phosphate buffer (pH 6.5) at 45°C for 5 min.

### Synthesis of Butyl Butyrate Using RmEstA

**Figure 5:**
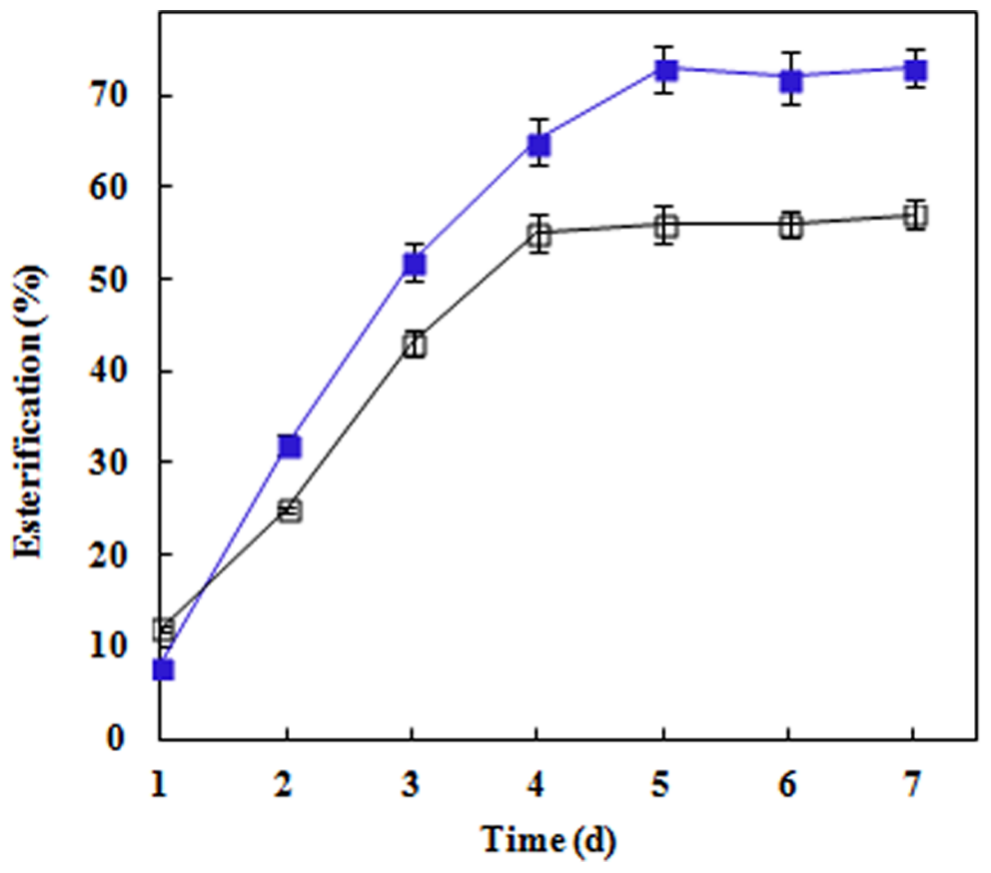
Temporal profile of butyl butyrate synthesis catalyzed by free (□) and immobilized (▪) RmEstA.

RmEstA in free form showed significant esterification efficiency (56%) in the synthesis of butyl butyrate from 1-butanol and butyric acid. However, when the free enzyme was immobilized on an AOT-based organogel, a 1.3-fold higher (73%) esterification efficiency was obtained for butyl butyrate synthesis (Figure 5). The optimal ratio of the two substrates for butyl butyrate synthesis was investigated. Holding the concentration of butyric acid constant at 100 mM and varying that of 1-butanol from 50–175 mM gave the highest esterification yield of 87% with 125 mM 1-butanol on day 5 (Table 5). 1-Butanol was then held at a constant 125 mM, and the concentration of butyric acid was varied from 50 to 175 mM. The highest esterification yield of 92% was achieved with 150 mM butyric acid (Table 5).

**Table 5:** Effect of substrate concentration on butyl butyrate synthesis by the immobilized esterase.

Substrate	Concentration (mM)	Esterification (%)
1-Butanol^a^	50	34±1.2
	75	56±2.4
	100	73±2.1
	125	87±3.6
	150	78±1.8
	175	55±1.2
Butyric acid^b^	50	44±1.5
	75	59±2.1
	100	82±2.8
	125	87±3.8
	150	92±3.3
	175	74±3.1

a The concentration of 1-butanol was varied from 50 to 175 mM, while the concentration of butyric acid was 100 mM.

b The concentration of butyric acid was varied from 50 to 175 mM, while the concentration of 1-butanol was 125 mM.

## Discussion

Lipolytic enzymes have been classified into eight families (families I to VIII) based on their conserved sequence motifs and biological properties [33]. Evidence for the existence of more additional lipolytic enzyme families, i.e. family IX [34], family X [35] and family XIII [36] has been further published. Family IX is also known as the HSL family since lipolytic enzymes from this family display a striking amino-acid-sequence similarity to the mammalian HSL [33]. To date, all reported microbial HSL esterases have been from bacteria, such as *Archaeoglobus fulgidus* [37], *Rheinheimera* sp. [38] and *Geobacillus thermodenitrificans* T2 [16]. RmEstA is the first reported fungal HSL esterase.

Although esterases are gaining attention for their wide range of applications, to date very few esterases have been characterized from fungi, in contrast to the number of esterases which have been characterized from bacteria [7]. Moreover, few fungal esterase genes have been cloned and expressed. Here we identified and cloned a novel esterase gene (*RmEstA*) from *R. miehei* CAU432, and successfully expressed it in *E. coli*. Fungi provide promising sources for the discovery of novel esterases. RmEstA is the first esterase to be characterized from a thermophilic fungus. The recombinant RmEstA was purified to homogeneity, with a molecular mass of 34 kDa as determined by SDS-PAGE (Figure 3), and a specific activity of 1,480 U mg^−1^ protein. The molecular mass of RmEstA is in accordance with most other reported microbial esterases, which range from 20 to 60 kDa [4], [12], [14], [17].

Most esterases show optimal activity at pH values above 7.0 and are stable in a narrow alkaline pH range [4], [12], [17]. RmEstA’s optimal pH of 6.5 (Figure 4A) is lower than that of most other reported esterases, such as from *Pseudoalteromonas arctica* (pH 7.5) [13], *Bacillus licheniformis* (pH 8.0) [9], *Pseudoalteromonas* sp. (pH 8.0) [4], *B. pseudofirmus* (pH 8.5) [14] and *Rhodococcus* sp. LKE-028 (pH 11.0) [10], but it is higher than that of the esterases from *Oenococcus oeni* (pH 5.0) [12] and *Pleurotus sapidus* (pH 6.0) [11]. RmEstA showed excellent stability across a broad range of pHs from 5.0 to 10.6 (Figure 4B), which is much wider than most other esterases [4], [9], [17], [30]. RmEstA exhibited maximal activity at 45°C, i.e. at a temperature that is lower than that of most other esterases from bacteria, including *B. pseudofirmus* (50°C) [36], *P. sapidus* (50°C) [11], *B. licheniformis* (60–65°C) [9], *Thermoanaerobacter tengcongensis* (70°C) [14] and *Rhodococcus* sp. LKE-028 (70°C) [10], comparable to that of esterases from *Pelagibacterium halotolerans* B2 (45°C) [39] and *O. oeni* (45°C) [12], and clearly higher than that of the cold-adapted esterases from *P. arctica* (25°C) and *Pseudoalteromonas* sp. (35°C) [13]. An enzyme with a relatively low optimal reaction temperature can potentially improve the efficiency of industrial processes that should be carried out at low temperatures, such as cheese ripening, and offers possible economic benefits through potential energy savings [13].

Substrate specificity of RmEstA was determined using *p*NP-linked esters of various acryl chain lengths (C_2_ to C_16_), triglycerides (C_2_ to C_10_) and olive oil (Table 3). The enzyme was most active on *p*NP-linked esters with a chain length of up to C_10_, whereas low activity was detected for substrates with longer chain lengths. Highest activity was also observed for *p*NP-linked esters and triglycerides with carbon chain lengths of C_6_ and C_4_, indicating that RmEstA is a typical esterase rather than a lipase, since esterases favor esters with carbon chain lengths shorter than 10 for hydrolysis, while lipase acts more efficiently on esters with long carbon chain lengths [3]. It should be noted that the esterase in the present study could hydrolyze *p*NP esters with chain lengths longer than C_14_, which is different from most other reported esterases, such as the enzymes from *Oenococcus oeni* [12], *Geobacillus thermodenitrificans* T2 [16] and *Rhodococcus* sp. LKE-028 [10]. The present esterase could also degrade olive oil, a property characteristic of lipases. The remarkably broad substrate specificity offers great potential for the use of this esterase in the production of a diverse range of high-value products.

*K*_m_ values were comparable to or lower than those of most bacterial esterases. An increase in chain length from C_2_ to C_4_ resulted in a slight decrease in the respective *K*_m_ values, and a significant increase in catalytic efficiency (*k*_cat_/*K*_m_). On the other hand, *V*_max_ values were not obviously affected, indicating a preference of RmEstA for *p*NP esters with a chain length of C_4_. However, the *K*_m_ values for both *p*NPB (C_2_) and *p*NPH (C_4_) were lower than those reported for other bacterial esterases [9], [10], [14].

Flavor compounds are one of the most important additives in today’s food industry, and biocatalysts play an important role in their synthesis [5]. Among the various enzymes, lipases have demonstrated unique properties that can be exploited for the synthesis of flavor esters [5], [6], [9], and several flavor esters, such as ethyl butyrate, ethyl acetate, ethyl isovalerate, isoamyl acetate and ethyl caprylate have been synthesized by various lipases [5], [6]. The esterase in the present study exhibited the same synthetic ability. RmEstA in free form converted butyric acid and 1-butanol to butyl butyrate with an esterification efficiency of 56%. When RmEstA was immobilized on an AOT-based organogel, the immobilized enzyme showed much higher esterification efficiency (92%) than the free enzyme (Table 5) after 7 days of incubation. Similar enhancement of ester synthesis by immobilized enzyme was also obtained with a lipase from *Burkholderia multivorans* [6]. This might have been due to immobilization of the enzyme on a support matrix which can improve the catalytic activity of the enzyme by providing protection against the inhibitory effect of organic solvents [6]. In addition, enzyme immobilization can cut enzyme costs by improving its reusability based on the easy recovery protocol in commercial use. The high esterification efficiency and reusability are very important from an economical point of view, making this enzyme attractive for possible practical application in the synthesis of flavor esters.
